# Editorial

**Published:** 1846-03

**Authors:** 


### THE MEDICAL EXAMINER.

PHILADELPHIA, MARCH, 1846.

### HEALTH OF PHILADELPHIA.

The inhabitants of the city of Philadelphia probably never enjoyed better health in the month of February than the present year. The small-pox, which has been more than usually prevalent, is rapidly disappearing, and we have reason to believe will shortly cease to exist. This arises, doubtless, from exhaustion of the materials for its continuance, in consequence of those subject to it having either had the disease or been vaccinated. Although there have been numerous instances of its occurrence in persons who had been vaccinated, as well as in some who had already had it by inoculation, and in the natural way, we believe we state the universal conviction of the profession when we assert, that the value of vaccination was never more triumphantly manifested than during the recent epidemic.

### TRANSYLVANIA UNIVERSITY.

By a slip from the Lexington Observer, we are informed that Dr. L. G. Watson has resigned the Chair of Theory and Practice of

Medicine in this Institution, and that Dr. Bartlett, the former occupant, has been appointed to fill the vacancy. Dr. Bartlett is now in Europe, but no doubt is entertained of his acceptance of the place.

Dr. Samuel Anan, of Baltimore, has been elected to the Chair of Obstetrics in the same school, vacated by the death of the late Professor Richardson.

On the resignation of Professor Watson, the class held a meeting, at which resolutions were passed, expressing, in appropriate language, their regret for the condition of the Professor's health, which rendered it necessary for him to retire, and their respect for him as a man and a teacher.

---

NEW MEDICAL SCHOOL.

The Legislature of Pennsylvania has recently chartered a new Medical College, to be located in Philadelphia, and called the "Franklin Medical College," of which Drs. Goddard, Clymer, Tucker, Rogers, Biddle, Van Wyck and Joynes, are to compose the Faculty.

---

NEW PUBLICATIONS.

Since the publication of our last number, in addition to those noticed in the present, we have received the following valuable works, which we shall notice more particularly hereafter, viz.: Velpeau's Operative Surgery, by Prof. Mott; Lectures on the Operations of Surgery, by Liston and Mütter; Liston's Elements of Surgery, with notes and additions, by Prof. Gross; Compendium of Lectures on the Theory and Practice of Medicine, delivered by Prof. Chapman, by Benedict; A Manual of Chemistry, by Hoblyn; Examinations in Anatomy, Physiology, Practice of Medicine, &c., by Hooper; Lecture on Medical Obedience, introductory to the course of Theory and Practice of Medicine in the Medical Department of Pennsylvania College, by William Darrach, M. D., etc.

---

ANNUAL REPORTS.

We have received the "*Twenty-fifth Annual Report of the Bloomingdale Asylum for the Insane. For the year* 1845." From Dr. P. Earl; and also,

The "Report of the Pennsylvania Hospital for the Insane, for the year 1845." From Dr. T. S. Kirkbride.

We have not had time to look into these "Reports," but feel assured that they lack none of the interest which characterized their predecessors. Will not some of our Correspondents furnish us with an analysis of these and others from kindred Institutions?

---

CATALOGUES.

We have before us the following Catalogues of Medical Students the present year:

| | | | | |
|---|---|---|---|---|
| "Western Reserve College,"—number of Medical Students, | | | | 160 |
| "University of Pennsylvania," | " | " | " | 462 |
| "Jefferson Medical College," | " | " | " | 469 |

